# Dynamic Ester-Linked Vitrimers for Reprocessable and Recyclable Solid Electrolytes

**DOI:** 10.3390/polym17141991

**Published:** 2025-07-21

**Authors:** Xiaojuan Shi, Hui Zhang, Hongjiu Hu

**Affiliations:** 1 Shanghai Institute of Applied Mathematics and Mechanics, Shanghai Key Laboratory of Mechanics in Energy Engineering, School of Mechanics and Engineering Science, Shanghai University, Shanghai 200072, China; hmingzhang007@163.com; 2 Shanghai Institute of Applied Mathematics and Mechanics, Shanghai Key Laboratory of Mechanics in Energy Engineering, Shanghai Frontier Science Center of Mechanoinformatics, School of Mechanics and Engineering Science, Shanghai University, Shanghai 200072, China

**Keywords:** polymer electrolyte, dynamic covalent ester bonds, reprocessable, recyclable

## Abstract

Traditional covalently cross-linked solid-state electrolytes exhibit desirable mechanical durability but suffer from limited processability and recyclability due to their permanent network structures. Incorporating dynamic covalent bonds offers a promising solution to these challenges. In this study, we report a reprocessable and recyclable polymer electrolyte based on a dynamic ester bond network, synthesized from commercially available materials. Polyethylene glycol diglycidyl ether (PEGDE) and glutaric anhydride (GA) were cross-linked and cured in the presence of benzyl dimethylamine (BDMA), forming an ester-rich polymer backbone. Subsequently, 1,5,7-triazabicyclo[4.4.0]dec-5-ene (TBD) was introduced as a transesterification catalyst to facilitate network rearrangement. Lithium bis(trifluoromethanesulfonyl)imide (LiTFSI) was incorporated to establish efficient ion transport pathways. By tuning the cross-linking density and catalyst ratio, the electrolyte achieved an ionic conductivity of 1.89 × 10^−5^ S/cm at room temperature along with excellent reprocessability.

## 1. Introduction

To address the increasing demand for safer and more sustainable lithium-ion batteries (LIBs), solid-state electrolytes (SSEs) have gained widespread attention as potential replacements for conventional liquid electrolytes [1,2,3,4]. Among these electrolytes, polymer-based SSEs are particularly promising due to their lightweight nature, structural tunability, processability, and ability to form conformal interfaces with electrodes [5,6,7]. Poly(ethylene oxide) (PEO) and its derivatives have served as foundational materials for polymer electrolytes, owing to their ability to solvate lithium salts and facilitate ion transport. Recent advancements have expanded the material landscape to include diverse polymer backbones and architectures [8,9,10]. Nevertheless, achieving high ionic conductivity at ambient temperature in solvent-free systems remains a significant challenge [11]. Moreover, the mechanical softness of linear or non-covalently cross-linked polymer electrolytes often leads to poor dimensional stability, especially under elevated temperatures or stack pressures required for battery operation. To address this issue, permanent covalent cross-linking strategies have been widely employed [12,13,14,15,16]. However, such permanent thermoset networks are inherently non-reprocessable, hindering reprocessing and limiting compatibility with large-scale battery manufacturing workflows. Additionally, the lack of recyclability in these materials poses environmental and economic concerns at the battery’s end of life, particularly given the increasing focus on circular material design and green electronics.

In recent years, dynamic covalent chemistry has emerged as a promising avenue for addressing these limitations [17,18,19,20,21,22]. By enabling reversible bond exchange under specific stimuli, dynamic covalent networks (known as vitrimers if the exchange reaction mechanism is associative) combine the structural integrity of thermosets with the adaptability of thermoplastics. This unique combination allows materials to be reprocessed, reshaped, or even recycled while maintaining their functional performance. For instance, Evans et al. developed a polyethylene oxide (PEO)-based network containing dynamic boronate cross-links to fabricate a recyclable solid polymer electrolyte (SPE). Although its room temperature ionic conductivity (25 °C) was limited to 1 × 10^−6^ S/cm, that study demonstrated the potential of dynamic network SPEs as a platform for recyclable lithium-ion battery electrolytes [20]. Subsequently, Lin et al. incorporated dynamic vinyl carbamate bonds into a lithium-containing PEO network electrolyte, achieving a polymer electrolyte with moderate ionic conductivity (~10^−5^ S/cm) alongside excellent reprocessability. Their studies revealed that lithium bis(fluorosulfonyl)imide (LiFSI) salt catalyzed bond exchange, enhancing network dynamics and enabling tunable properties through reagent composition adjustments [21]. Ullah et al. synthesized an SPE based on a PEO network, cross-linked with dynamic disulfide bonds and cage-like polyhedral oligomeric silsesquioxane (POSS). The dynamic disulfide bonds endowed the SPE with good recyclability and reprocessability, while the POSS enhanced the cross-linking density and mechanical properties of the material. Despite its modest ionic conductivity (~10^−5^ S/cm at 27 °C), the healed and recycled electrolytes retained conductivity comparable with that of the original samples [22]. However, these systems often rely on specialized or synthetically demanding components, and comprehensive studies on scalable, high-performance dynamic networks using readily available materials remain limited.

In this work, we report a reprocessable and recyclable solid-state polymer electrolyte featuring a dynamic ester bond network constructed from commercially available components. The network is formed via the curing of polyethylene glycol diglycidyl ether (PEGDE) with glutaric anhydride (GA) in the presence of benzyl dimethylamine (BDMA), resulting in a robust, ester-rich matrix. A transesterification catalyst, 1,5,7-triazabicyclo[4.4.0]dec-5-ene (TBD), is subsequently introduced to enable dynamic bond exchange and network rearrangement. Lithium bis(trifluoromethanesulfonyl)imide (LiTFSI) is incorporated to facilitate efficient ion transport. Through systematic tuning of the network structure and catalyst content, the resulting polymer electrolyte exhibits notable ionic conductivity at room temperature (~10^−5^ S/cm), as well as excellent mechanical properties and thermal reprocessability. This work provides a scalable and sustainable strategy for designing next-generation polymer electrolytes with enhanced processability and end-of-life recyclability.

## 2. Experiments

### 2.1. Preparation of Ester-Linked Vitrimer Electrolytes

The chemicals used in this study were polyethylene glycol diglycidyl ether (PEGDE), glutaric anhydride (GA), LiTFSI, and TBD, which were procured from Shanghai Macklin Biochemical Co., Ltd. (Shanghai, China), while N, N-dimethylbenzylamine (BDMA) was acquired from Shanghai Aladdin Biochemical Technology Co., Ltd. (Shanghai, China). No additional purification was performed on any of the chemicals prior to their use in the experiments.

The ester-linked vitrimer electrolytes (ELVEs) were synthesized as follows: BDMA (5 mol%), TBD (5 mol%), LiTFSI, PEGDE, and GA were combined in reagent bottles. The mixture was homogenized at 100 °C using a mechanical homogenizer until a uniform solution was obtained. The obtained precursor solution was poured onto pre-cleaned glass substrates or polytetrafluoroethylene (PTFE) molds. After vacuum drying at 100 °C for 30 min, the samples were subjected to stepwise curing at 150 °C for 2 h under atmospheric pressure, resulting in the formation of the ester-linked vitrimer electrolytes. During synthesis, BDMA acts as a nucleophilic curing accelerator, promoting the ring-opening polymerization of epoxy groups to form a stable cross-linked network [23]. TBD serves as a strong organic base catalyst, enabling dynamic transesterification reactions that impart vitrimer behavior through reversible covalent bond exchange [24].

To explore the correlation between LiTFSI mass fraction and ionic conductivity, a series of samples were prepared with a fixed epoxy-to-carbonyl molar ratio of 1.5:1 while varying the LiTFSI loading (0.5, 1, 1.5, 2, 2.5, 3, and 4 wt%). To examine the influence of cross-linking density, additional samples were prepared with a constant LiTFSI content of 2.5 wt% by adjusting the epoxy-to-carbonyl molar ratios to 1.25:1, 1.4:1, 1.5:1, 1.6:1, and 1.75:1.

### 2.2. Reprocessing of Ester-Linked Vitrimer Electrolytes

The ELVE films were thermally rejoined under controlled conditions (150 °C for 2–10 h) to weld the vitrimer electrolyte membranes. The welding process involved the reformation of interfacial bonds through thermally activated dynamic covalent interactions. To quantify changes in bonding efficiency, the rejoined films were subjected to uniaxial tensile testing using a DMA Q800 system (TA Instruments, New Castle, DE, USA) under consistent parameters (10%/min strain rate, 25 °C). The selected strain rate of 10%/min ensures consistent deformation while minimizing stress concentration [19,25,26,27].

### 2.3. Characterization

Fourier-transform infrared (FTIR) spectroscopy was performed on a Nicolet iS50 spectrometer (Thermo Scientific, Waltham, MA, USA) with spectral acquisition spanning 4000–600 cm^−1^. Thermal transitions were analyzed using differential scanning calorimetry (DSC Q2000, TA Instruments, New Castle, DE, USA) under a nitrogen purge (50 mL/min flow rate). Samples were subjected to a temperature ramp from −80 °C to 80 °C at 10 °C/min to determine the glass transition temperature (*T_g_*). A thermogravimetric analysis (TGA Q50, TA Instruments, New Castle, DE, USA) was conducted by heating specimens to 500 °C at 10 °C/min under a nitrogen atmosphere. Mechanical properties were evaluated via uniaxial tensile testing on a dynamic mechanical analyzer (DMA Q800, TA Instruments, New Castle, DE, USA) in static mode. The ELVE strips were stretched at a constant strain rate of 10%/min until fracture, and stress–strain curves were recorded for modulus calculations. Stress relaxation experiments were conducted on the ELVE strips using the stress relaxation mode of the same DMA Q800 at temperatures ranging from 80 to 130 °C. The heating rate was set to 10 °C/min, and the strain was fixed at 0.5%. The ionic conductivity of the electrolyte was measured via electrochemical impedance spectroscopy (EIS) at temperatures ranging from 25 to 105 °C. Measurements were carried out using a Zennium Pro electrochemical workstation (Zahner, Kronach, Germany) over a frequency range of 1 MHz to 0.1 Hz. The film was placed between two symmetric stainless-steel electrodes (diameter: 15.8 mm).

## 3. Results and Discussion

### 3.1. Synthesis and Characterization of Ester-Linked Vitrimer Electrolytes

**Figure 1:**
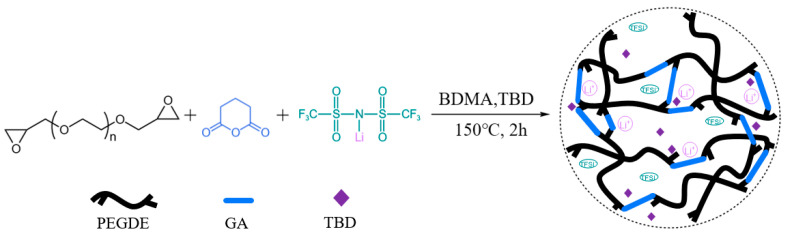
Synthetic routes of ester-linked vitrimer electrolytes.

The synthetic route for the ester-linked vitrimer electrolytes is illustrated in Figure 1. BDMA was used as the initiator in the curing process, and TBD served as the transesterification catalyst. In the presence of BDMA and TBD, the epoxy groups of PEGDE react with the carbonyl groups of GA to form a cross-linked polymer network containing dynamic covalent ester bonds. Simultaneously, LiTFSI is incorporated to establish ion conduction pathways, resulting in an ester vitrimer electrolyte with both structural reconfigurability and efficient ion transport.

**Figure 2:**
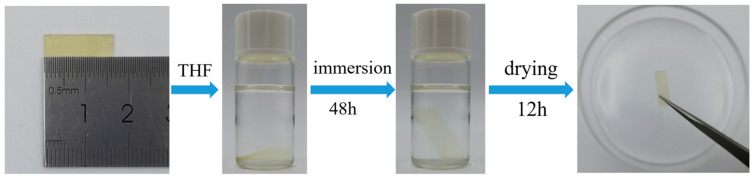
Gel fraction measurements of the ELVE samples.

As illustrated in Figure 2, the gel fraction analysis of the ELVE using tetrahydrofuran (THF) revealed a highly insoluble fraction exceeding 97.76% (±0.34%), confirming the formation of a robust cross-linked architecture. This result is consistent with the presence of a densely interconnected polymer network.

**Figure 3:**
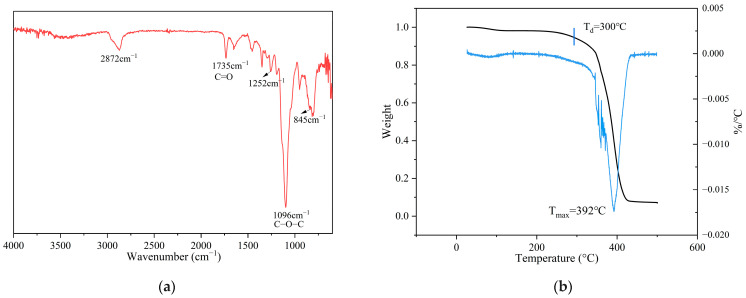
(**a**) FTIR spectra of the ELVE; (**b**) TGA (black line) and DTG (blue line) curves of the ELVE.

As indicated by the FTIR spectra (Figure 3a), the formation of the polymer network is clearly confirmed. The characteristic peak at 2872 cm^−1^ originates from the stretching vibration of –CH_2_– groups in the polyethylene glycol (PEG) segments. The characteristic epoxy absorption bands appear at 845 cm^−1^ and 1252 cm^−1^. In addition, the C–O–C ether bond stretching vibration appears at 1096 cm^−1^. A distinct carbonyl (C=O) stretching band at 1735 cm^−1^ further confirms the formation of ester linkages. Thermal stability, a critical factor for battery safety and longevity [28], was then evaluated via TGA (Figure 3b, black line) and DTG (Figure 3b, blue line). The initial decomposition temperature (*T*_d_) was around 300 °C, with the DTG peak indicating an actual decomposition temperature of 392 °C. The TGA and DTG curves show a two-step thermal degradation: a gradual weight loss beginning at ~300 °C attributed to ester bond breakdown, followed by a sharp decline between 300 and 400 °C associated with polymer backbone degradation. These thermal behaviors are consistent with those reported for vitrimeric systems [29], confirming the electrolyte’s exceptional thermal resilience under high-temperature conditions.

**Figure 4:**
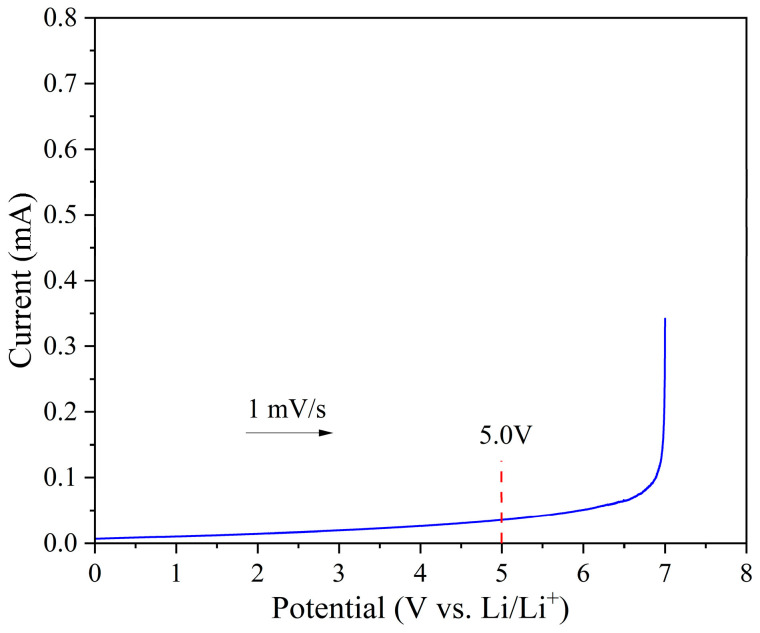
The electrochemical stability window of the ELVE.

The electrochemical window is a key parameter for evaluating the electrochemical stability of electrolytes. A wider electrochemical window indicates broader applicability and better compatibility with high-voltage electrodes. Therefore, linear sweep voltammetry (LSV) was conducted using an electrochemical workstation to assess the electrochemical stability of the ELVE. Stainless steel (SS) was used as the working electrode and lithium metal as the reference electrode, with the voltage swept from 0 V to 7 V at a scan rate of 1 mV/s at 60 °C. The resulting electrochemical window is shown in Figure 4. The oxidative decomposition potential of the electrolyte is around 5 V, indicating good electrochemical stability and the potential applicability of the ELVE as a solid-state electrolyte for lithium metal batteries.

### 3.2. Electrochemical Performance of the Ester-Linked Vitrimer Electrolytes

**Figure 5:**
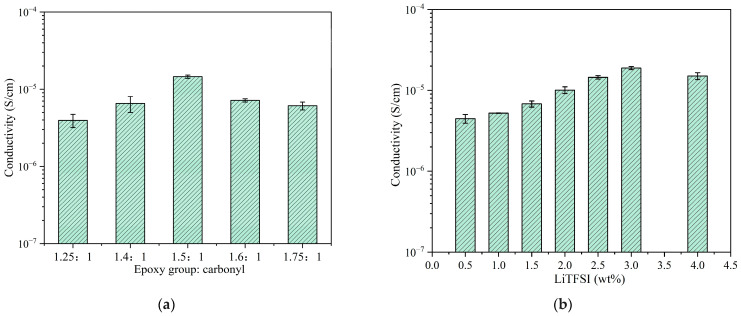
(**a**) Ionic conductivity of the ELVE with different cross-linking ratios at 25 °C; (**b**) ionic conductivity of the ELVE with different lithium salt concentrations.

The cross-linking ratio of epoxy to carbonyl groups and the lithium salt content in the ELVE were optimized to achieve higher ionic conductivity. As shown in Figure 5a, within the epoxy-to-carbonyl ratio range of 1.25–1.75, the ionic conductivity of the ELVE sample initially increases and then declines as the epoxy group content rises, reaching a maximum of 1.45 × 10^−5^ S/cm at an epoxy-to-carbonyl ratio of 1.5. The initial increase is attributed to the enhanced network flexibility resulting from a higher PEGDE content, which facilitates polymer chain motion and thus improves ionic conductivity [30,31]. In addition, the DSC results (Figure S1) show that the glass transition temperature (*T_g_*) decreases with increasing PEGDE content. This decrease in *T_g_* is attributed to the increased flexibility of the network, which further supports the above conclusion. However, excessive epoxy groups lead to over-coordination of Li^+^ by ether oxygen atoms, reducing Li^+^ mobility [32,33,34,35]. Following the determination of the optimal cross-linking ratio, the lithium salt content was further optimized. As shown in Figure 5b, the ionic conductivity increases with the salt mass fraction, up to 3 wt%, reaching a peak of 1.89 × 10^−5^ S/cm, and then decreases at higher concentrations. The initial increase is attributed to the suppression of crystallinity and the increase in lithium salt concentration. The subsequent decrease is likely due to the interchain coordination induced by lithium ions [21,26,36]. Thus, the optimal formulation is determined to be an epoxy-to-carbonyl ratio of 1.5 and a lithium salt content of 3 wt%, yielding a room temperature ionic conductivity of 1.89 × 10^−5^ S/cm. This value is moderate compared with that for existing solid polymer electrolytes (SPEs) and gel polymer electrolytes (Table S2) [7,19,20,21,22,37,38,39,40,41].

**Figure 6:**
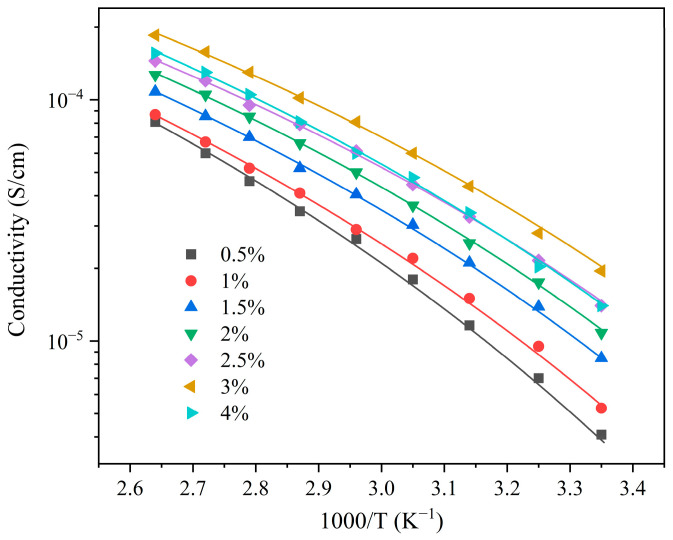
Temperature-dependent ionic conductivity at different lithium salt concentrations (symbols: experimental data; curves: VTF fitting).

The temperature-dependent ionic conductivity of the ELVE was investigated at various lithium salt concentrations (0.5–4 wt%) over the temperature range of 25–105 °C. As shown in Figure 6, the temperature-dependent ionic transport behavior exhibits a good fit with the Vogel–Tammann–Fulcher (VTF) model, which describes the segmental motion-coupled ion migration within the solid network [42,43,44]. The relationship between ionic conductivity (*σ*) and temperature (*T*) is given by Equation (1) as follows:

(1) $$\sigma =A\exp\left[-\frac{E_{a}}{R(T-T_{0})}\right]$$

where *A* is the pre-exponential factor, *E_a_* denotes the apparent activation energy, *R* represents the gas constant (8.315 J·mol^−1^·K^−1^), and *T*_0_ is the ideal glass transition temperature. Typically, *T*_0_ is about 50 K lower than *T_g_*, which is determined via DSC (Figure S2). The corresponding fitting parameters are summarized in Table 1. The calculated activation energy for ionic conduction in the ELVE with varying lithium salt concentrations ranges from 6.27 to 7.98 kJ/mol, which are typical values for Li salt-based electrolytes (Table S3) [18,35,45,46].

### 3.3. Recyclability and Reprocessability of the Ester-Linked Vitrimer Electrolytes

**Figure 7:**
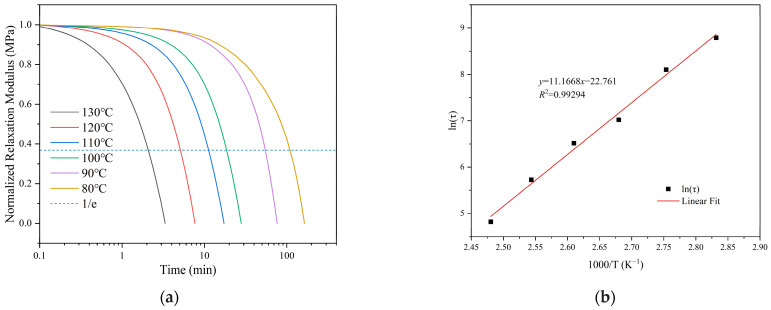
(**a**) Stress-relaxation curves of the ELVE at various temperatures; (**b**) Arrhenius plot of the characteristic relaxation time as a function of inverse temperature.

The recyclability of the ELVE stems from the dynamic ester bonds, which enable reversible exchange within the polymer network. To quantitatively investigate dynamic behavior and evaluate reprocessability, stress relaxation experiments were performed between 80 °C and 130 °C. As illustrated in Figure 7a, the internal stress gradually dissipates over time, confirming that the polymer chains are linked by reversible bonds and undergo substantial network rearrangement under thermal conditions. Dynamic polymer networks are commonly described using the Maxwell model, in which stress relaxation is characterized by a single relaxation time constant (*τ*), defined as the time required for the relaxation modulus to decay to 1/e (approximately 36.8%) of its initial value [45,46] (Table S1). Furthermore, the relaxation time exhibited a clear temperature dependence, which conformed well to the Arrhenius relationship (Figure 7b). The Arrhenius analysis using Equation (2) provided an activation energy *E_a_* of 93.7 kJ/mol for the ELVE vitrimer. This enhanced responsiveness at elevated temperatures is attributed to accelerated transesterification reactions within the dynamic network, facilitating efficient stress dissipation and enabling practical reprocessing and recycling as follows:

(2) $$\tau =\tau_{0}\exp\left(-\frac{E_{a}}{RT}\right)$$

where *τ* is the relaxation time constant, *τ*_0_ denotes the pre-exponential factor, *R* represents the gas constant (8.315 J·mol^−1^·K^−1^), and *T* is the absolute temperature.

**Figure 8:**
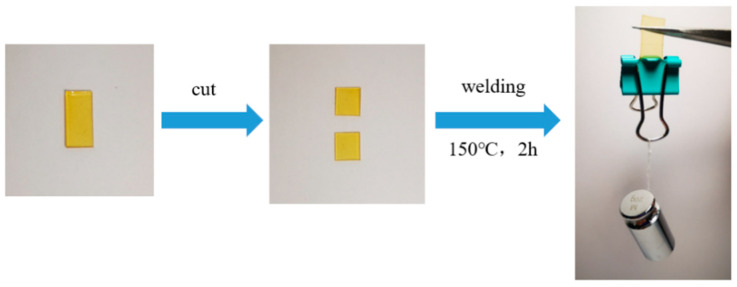
Electrolyte repair experiment.

To demonstrate the recyclability of the prepared ELVE, the samples were cut and reassembled. The cut segments were gently aligned and pressed under moderate pressure (by placing a 50 g weight, approximately 0.5 N, at the interface) to ensure good interfacial contact, followed by heat bonding at 150 °C for 2 h. The recycled film exhibited sufficient mechanical integrity and was capable of supporting a 20 g weight without breaking (Figure 8).

**Figure 9:**
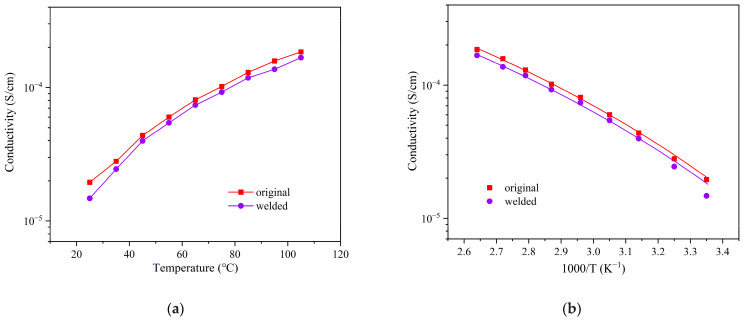
(**a**) Ionic conductivity of the original and welded ELVE samples across a range of temperatures (25–105 °C); (**b**) VTF fitting of the temperature-dependent ionic conductivity for the original and welded ELVE samples.

Impedance measurements revealed that the reprocessed ELVE sample retained ionic conductivity similar to that of the original sample, indicating that dynamic network reorganization effectively preserves continuous ion transport pathways and confirms good recyclability (Figure 9a). Furthermore, the pristine and recycled ELVE samples exhibited similar temperature-dependent ionic conductivity, both of which are well described by the VTF model (Figure 9b), with calculated activation energies of 6.27 kJ/mol and 6.25 kJ/mol, respectively.

**Figure 10:**
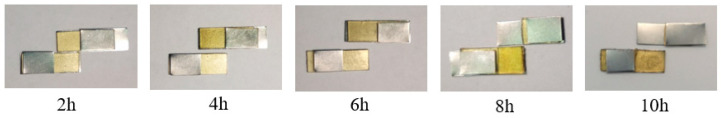
Fracture morphology after welding.

To further evaluate the welding efficacy, mechanical tests were conducted on the ELVE samples subjected to various welding durations (2, 4, 6, 8, and 10 h). As shown in Figure 10, fractures initially occurred at the repaired interface for shorter welding times (2–6 h), whereas specimens welded for 8 and 10 h fractured within the bulk material, indicating restored bulk strength.

**Figure 11:**
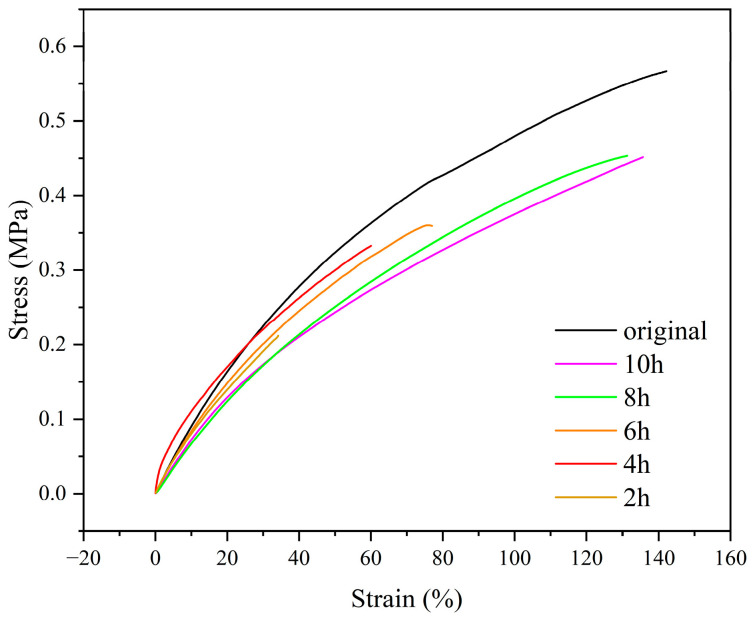
Evolution of the mechanical strength of the ELVE samples with welding time.

The stress–strain curves of the welding ELVE revealed progressive mechanical recovery with extended welding durations (Figure 11). The pristine ELVE exhibited a tensile strength of 0.58 ± 0.02 MPa, a Young’s modulus of 1.00 ± 0.06 MPa, and a toughness of 0.51 ± 0.01 MPa, indicating good mechanical performance suitable for practical applications. After 8 h of welding, the reprocessed ELVE showed a reduction in mechanical properties, with a tensile strength of 0.44 ± 0.02 MPa, a Young’s modulus of 0.57 ± 0.40 MPa, and a toughness of 0.34 ± 0.03 MPa. This reduction in performance is likely attributed to lithium-catalyzed side reactions, which degrade the polymer network [21]. Nevertheless, it retained sufficient structural integrity to function effectively as a separator.

## 4. Conclusions

In summary, a reprocessable and recyclable solid-state polymer electrolyte was successfully developed by incorporating dynamic covalent ester bonds into the polymer network. This design addresses the inherent limitations of traditional covalently cross-linked electrolytes, enabling scalable fabrication and end-of-life recyclability without compromising mechanical integrity or ionic conductivity. The dynamic network structure facilitates thermal reprocessing and mechanical recovery. These results highlight the potential of dynamic covalent polymer networks to advance both sustainable and high-performance solid-state battery technologies. To further enhance ionic conductivity, future efforts will focus on strategies such as side-chain modification, plasticizer, or PEG/ionic liquid blending and nanofiller addition to improve ion transport, chain mobility, and interfacial dynamics.

## Figures and Tables

**Table 1:** Vogel–Tammann–Fulcher parameters of the ELVE at different lithium salt concentrations.

LiTFSI (wt%)	*A* (S/cm)	*T_0_* (K)	*E_a_* (kJ/mol)	*T_g_* (K)
0.5%	8.76 × 10^−3^	174	7.98	224
1.0%	6.76 × 10^−3^	172	7.49	222
1.5%	6.12 × 10^−3^	171	6.97	221
2.0%	6.48 × 10^−3^	170	6.79	220
2.5%	5.80 × 10^−3^	170	6.38	220
3.0%	6.91 × 10^−3^	169	6.27	219
4.0%	7.97 × 10^−3^	168	6.86	218

## Data Availability

The data are contained within this article.

## Supplementary Materials

The following supporting information can be downloaded at https://www.mdpi.com/article/10.3390/polym17141991/s1; Figure S1: DSC curves of ELVE at different cross-linking ratios; Figure S2: DSC curves of ELVE at different lithium salt contents; Table S1: Corresponding time constants (τ) at different temperatures; Table S2: Room temperature ionic conductivity of dynamic/vitrimer-based and conventional solid polymer or gel polymer electrolytes; Table S3: Activation energy (*E*_a_) of different lithium salt electrolytes. References [7,18,19,20,21,22,35,37,38,39,40,41,45,46] are cited in the supplementary materials.
