# Supplementary material for: Dynamic Ester-Linked Vitrimers for Reprocessable and Recyclable Solid Electrolytes
Source: Polymers (Basel). 2025 Jul 21;17(14):1991. doi: 10.3390/polym17141991 (PMC12298296; doi:10.3390/polym17141991)
Supplement: Supplementary file 1 [file polymers-17-01991-s001.zip › polymers-3739462-supplementary.pdf]

polymers

Supplementary Material

Dynamic Ester-Linked Vitrimers for Reprocessable and Recyclable Solid Electrolytes

Xiaojuan Shi [1,2,*], Hui Zhang [1] and Hongjiu Hu [1,2,*]

[1] Shanghai Institute of Applied Mathematics and Mechanics, Shanghai Key Laboratory of Mechanics in Energy Engineering, School of Mechanics and Engineering Science, Shanghai University, Shanghai 200072, China; hmingzhang007@163.com
[2] Shanghai Institute of Applied Mathematics and Mechanics, Shanghai Key Laboratory of Mechanics in Energy Engineering, Shanghai Frontier Science Center of Mechanoinformatics, School of Mechanics and Engineering Science, Shanghai University, Shanghai 200072, China
* Correspondence: xiaojuan-shi@shu.edu.cn (X.S.); huhongjiu@shu.edu.cn (H.H.)

DSC analysis showed that the glass transition temperatures (T_g) of ELVE with epoxy-to-carbonyl molar ratios of 1.25:1, 1.4:1, 1.5:1, 1.6:1, and 1.75:1 were –49 °C, –51 °C, –53 °C, –53 °C, and –55 °C, respectively.

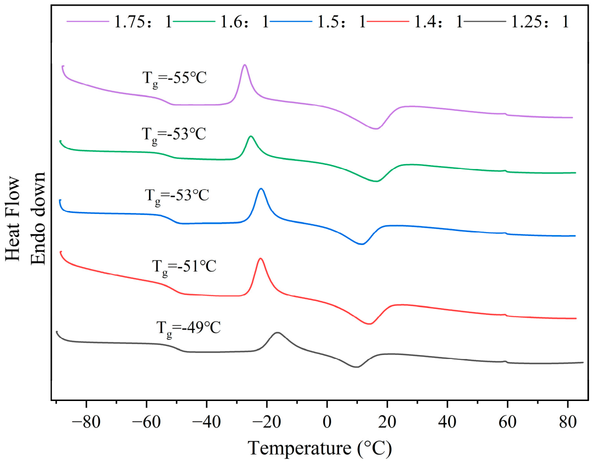

Figure S1. DSC curves of ELVE at different cross-linking ratios.

DSC analysis showed that the T_g values of ELVE containing 0.5%, 1.0%, 1.5%, 2.0%, 2.5%, 3.0%, and 4.0% lithium salt were -49°C, -51°C, -52°C, -53°C, -53°C, -54°C, and -55°C, respectively.

 https://doi.org/10.3390/polym17141991

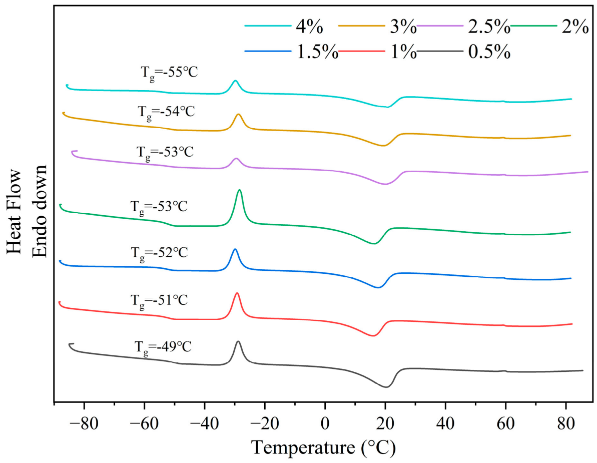

Figure S2. DSC curves of ELVE at different lithium salt contents.

Table S1. Corresponding time constants (τ) at different temperatures.

Temperature	τ (s)
130 °C	124.2
120 °C	307.2
110 °C	676.2
100 °C	1119.0
90 °C	3296.4
80 °C	6571.2

Table S2. Room temperature ionic conductivity of dynamic/vitrimer-based and conventional solid polymer or gel polymer electrolytes.

Polymer electrolytes	*Ionic conductivity (S/cm)*	Refs.
V-SPE-2000	3.30×10^{-5} S/cm (30°C)	[1]
Dynamic network electrolytes	~10^{-6} S/cm (25°C)	[2]
Vitrimer electrolytes	~10^{-5} S/cm (25°C)	[3]
P(TU-EO)-POSS	2.1×10^{-5} S/cm (300K)	[4]
PPBSPE	~10^{-5} S/cm (30°C)	[5]
SHSPEs (PEGSS-PEGDMA)	8.96×10^{-6} S/cm (30°C)	[6]
PBAD-PEG	8.67×10^{-4} S/cm(25°C)	[7]
DEC electrolyte	6×10^{-4} S/cm(25°C)	[8]
PEO-SPE-TPC	~10^{-5} S/cm (30°C)	[9]
PEO-DVB-PEGDA	3.0×10^{-6} S/cm (25°C)	[10]
ELVE	1.89×10^{-5} S/cm (25°C)	This work

Entries 1–5: vitrimer electrolytes; 7–8: gel electrolytes; 9–10: conventional electrolytes.

Table S3. Activation energy (E_a) of different lithium salt electrolytes.

Lithium Salt Electrolytes	E_a (kJ/mol)	Refs.
IBshPE ($LiPF_6$/LiDFOB/LiFSI)	13.8	[11]
PEG-co-PDSN ($LiClO_4$)	10.25~14.78	[12]
SIPEs ($LiClO_4$/LiDFOB)	10.9	[13]
PU-CA (LiBOB)	2.18~2.48	[14]
ELVE (LiTFSI)	6.27~7.98	This work

References

1. Yang, S.; Park, S.; Kim, S.; Kim, S.-K. Vitrimer with Dynamic Imine Bonds as a Solid-State Electrolyte for Lithium Metal Batteries. *Mater. Today Energy* **2024**, *45*, 101690. https://doi.org/10.1016/j.mtener.2024.101690.
2. Jing, B.B.; Evans, C.M. Catalyst-Free Dynamic Networks for Recyclable, Self-Healing Solid Polymer Electrolytes. *J. Am. Chem. Soc.* **2019**, *141*, 18932–18937. https://doi.org/10.1021/jacs.9b09811.
3. Lin, Y.; Chen, Y.; Yu, Z.; Huang, Z.; Lai, J.-C.; Tok, J.B.-H.; Cui, Y.; Bao, Z. Reprocessable and Recyclable Polymer Network Electrolytes via Incorporation of Dynamic Covalent Bonds. *Chem. Mater.* **2022**, *34*, 2393–2399. https://doi.org/10.1021/acs.chemmater.1c04396.
4. Ullah, S.; Wang, H.; Hang, G.; Zhang, T.; Li, L.; Zheng, S. Poly(Thiourethane-Co-Ethylene Oxide) Networks Crosslinked with Disulfide Bonds: Reinforcement with POSS and Use for Recyclable Solid Polymer Electrolytes. *Polymer* **2023**, *284*, 126318. https://doi.org/10.1016/j.polymer.2023.126318.
5. Wan, L.; Tan, X.; Du, X.; Xue, X.; Tong, Y.; Zhou, D.; Ling, Y.; Xie, Y.; Zhao, J. Self-Healing Polymer Electrolytes with Dynamic-Covalent Borate for Solid-State Lithium Metal Batteries. *Eur. Polym. J.* **2023**, *195*, 112191. https://doi.org/10.1016/j.eurpolymj.2023.112191.
6. Jo, Y.H.; Li, S.; Zuo, C.; Zhang, Y.; Gan, H.; Li, S.; Yu, L.; He, D.; Xie, X.; Xue, Z. Self-Healing Solid Polymer Electrolyte Facilitated by a Dynamic Cross-Linked Polymer Matrix for Lithium-Ion Batteries. *Macromolecules* **2020**, *53*, 1024–1032. https://doi.org/10.1021/acs.macromol.9b02305.
7. Zhou, S.; Wang, X.; Xu, Z.; Guan, T.; Mo, D.; Deng, K. Rapid Self-Healing, Highly Conductive and near-Single-Ion Conducting Gel Polymer Electrolytes Based on Dynamic Boronic Ester Bonds for High-Safety Lithium Metal Batteries. *J. Energy Storage* **2024**, *75*, 109712. https://doi.org/10.1016/j.est.2023.109712.
8. Yu, L.; Zhu, S.; Jiang, Z.; Tang, X.; Tian, T.; Hu, Z.; Du, P.; Wang, Y.; Tang, H. Dual In-Situ Curing Gel Polymer Electrolyte for Solid-State Lithium Battery. *Mater. Today Commun.* **2025**, *45*, 112414. https://doi.org/10.1016/j.mtcomm.2025.112414.
9. Sun, H.; Yang, Q.; Kong, D.; Li, Y.; He, Y.; Zhang, N.; Hu, H. Effect of Thermal Pre-compressing on Ionic Conductivity and Mechanical Properties of PEO -based Solid-state Electrolytes. *J. Appl. Polym. Sci.* **2024**, *141*, e56156. https://doi.org/10.1002/app.56156.
10. Ben Youcef, H.; Garcia-Calvo, O.; Lago, N.; Devaraj, S.; Armand, M. Cross-Linked Solid Polymer Electrolyte for All-Solid-State Rechargeable Lithium Batteries. *Electrochim. Acta* **2016**, *220*, 587–594. https://doi.org/10.1016/j.electacta.2016.10.122.
11. Zhou, S.; Deng, K.; Xu, Z.; Xiao, M.; Meng, Y. Highly Conductive Self-Healing Polymer Electrolytes Based on Synergetic Dynamic Bonds for Highly Safe Lithium Metal Batteries. *Chem. Eng. J.* **2022**, *442*, 136083. https://doi.org/10.1016/j.cej.2022.136083.
12. Nguyen, T.D.; Roh, S.; Nguyen, M.T.N.; Nam, Y.; Kim, D.-J.; Lim, B.; Yoon, Y.S.; Lee, J.S. Synthesis of a Copolymer with a Dynamic Disulfide Network and Its Application to a Lithium-Ion Capacitor Polymer Electrolyte. *Chem. Eng. J.* **2024**, *497*, 154430. https://doi.org/10.1016/j.cej.2024.154430.
13. Bin Rusayyis, M.A.; Torkelson, J.M. Reprocessable Covalent Adaptable Networks with Excellent Elevated-Temperature Creep Resistance: Facilitation by Dynamic, Dissociative Bis(Hindered Amino) Disulfide Bonds. *Polym. Chem.* **2021**, *12*, 2760–2771. https://doi.org/10.1039/D1PY00187F.
14. Zhang, L.; Chen, L.; Rowan, S.J. Trapping Dynamic Disulfide Bonds in the Hard Segments of Thermoplastic Polyurethane Elastomers. *Macro Chem. Phys.* **2017**, *218*, 1600320. https://doi.org/10.1002/macp.201600320.

Disclaimer/Publisher's Note: The statements, opinions and data contained in all publications are solely those of the individual author(s) and contributor(s) and not of MDPI and/or the editor(s). MDPI and/or the editor(s) disclaim responsibility for any injury to people or property resulting from any ideas, methods, instructions or products referred to in the content.
